# Electrocatalytic Innovations at Atomic Scale: From Single-Atom to Periodic Ensembles for Sustainable Energy Conversion

**DOI:** 10.3390/nano15080634

**Published:** 2025-04-21

**Authors:** Longlu Wang, Yang Liu

**Affiliations:** College of Electronic and Optical Engineering & College of Flexible Electronics (Future Technology), Nanjing University of Posts and Telecommunications, Nanjing 210023, China; 1223024939@njupt.edu.cn

**Keywords:** atomically dispersed catalysts, single-atom catalysts, dual-atom catalysts, periodic single-metal site catalysts

## Abstract

Atomically dispersed catalysts, including single-atom, dual-atom, and periodic single-metal site catalysts, have revolutionized electrocatalysis by merging atomic precision with heterogeneous stability. This review traces their evolution from pioneering stabilization strategies to advanced microenvironment engineering, enabling breakthroughs in oxygen reduction, hydrogen evolution, and CO_2_ reduction. SACs maximize atom utilization but face multi-step reaction limits, addressed by DACs through synergistic dual-site mechanisms. PSMSCs further enhance activity via ordered atomic arrangements, ensuring uniform active sites and mechanistic clarity. Key breakthroughs include microenvironment engineering to tailor active sites, as well as advanced characterization techniques revealing dynamic restructuring under operando conditions. The transition from isolated atoms to ordered ensembles highlights the importance of atomic-level control in unlocking new catalytic mechanisms. This work underscores the transformative potential of ADCs in sustainable energy technologies and provides a roadmap for future research in rational catalyst design, dynamic behavior analysis, and scalable synthesis.

## 1. Introduction

The global energy and environmental crisis, driven by climate change, fossil fuel depletion, and escalating pollution, demands urgent transitions to sustainable energy systems. Electrocatalytic technologies, such as fuel cells and CO_2_ conversion devices, are pivotal to this transition [1]. However, their efficiency and scalability heavily rely on high-performance catalysts. Traditional heterogeneous catalysts, while robust, suffer from low atom utilization and poorly defined active sites, whereas homogeneous catalysts lack stability and recyclability. Bridging this gap requires innovations that combine atomic precision with practical durability [2].

Atomically dispersed catalysts (ADCs) have emerged as a paradigm-shifting solution, offering near-theoretical atom efficiency, tunable active sites, and unique electronic properties. Since the landmark discovery of single-atom Pt catalysts in 2011, the field has rapidly evolved [3]. Single-atom catalysts (SACs) redefine catalytic boundaries by maximizing metal utilization and enabling atomic-level mechanistic studies [4]. Dual-atom catalysts (DACs) further enhance reactivity through synergistic interactions between paired metal sites, breaking linear scaling relationships in multi-step reactions [5]. Most recently, periodic single-metal site catalysts (PSMSCs) have introduced ordered atomic arrangements, merging the benefits of SACs and DACs while addressing challenges like inhomogeneous microenvironments and metal atom aggregation [6].

This review chronicles the development of ADCs, from early stabilization strategies to cutting-edge microenvironment engineering. We analyze breakthroughs in synthesis (e.g., defect engineering, atomic layer deposition, and thermal printing), structural modulation (e.g., strain, curvature, and periodic arrays), and operando characterization. By dissecting the interplay between atomic configuration, electronic structure, and catalytic performance, this work elucidates design principles for SACs, DACs, and PSMSCs. Furthermore, it highlights their transformative applications in energy conversion, including hydrogen production, CO_2_ valorization, and fuel cell technologies. Finally, we outline future directions, emphasizing the need for predictive modeling, dynamic stability studies, and industrial-scale fabrication. As the quest for sustainable energy intensifies, ADCs stand at the forefront of next-generation catalyst innovation, offering solutions to both fundamental and applied challenges in green chemistry.

## 2. Single-Atom Catalyst

**Figure 1:**
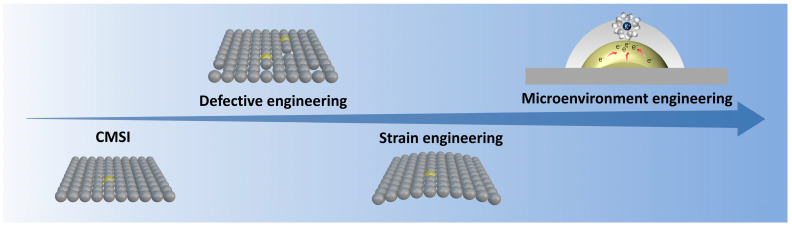
Schematic development of single atom catalysts.

SACs represent a valuable bridge between homogeneous and heterogeneous catalysis, offering significant advantages such as high atom utilization, an abundance of active sites, excellent catalytic activity and selectivity, and cost efficiency. The development of single-atom catalysts (SACs) has been driven by the need to overcome intrinsic limitations such as low stability, aggregation-prone metal atoms, and insufficient control over active site microenvironments (Figure 1). Early efforts focused on stabilizing isolated metal atoms through strong interactions with supports. In 2011, Qiao et al. [3] demonstrated that Pt atoms could be anchored on FeO_x_ via co-precipitation, leveraging metal–support interactions (MSIs) to prevent aggregation. This Pt_1_/FeO_x_ catalyst exhibited exceptional activity for CO oxidation, but its low Pt loading (<1 wt%) and reliance on defect sites highlighted the challenge of scalability. The absence of Pt–Pt bonds, confirmed by EXAFS and HAADF-STEM, underscored the potential of SACs but also exposed the need for more robust stabilization mechanisms beyond defect-dependent anchoring.

A pivotal breakthrough came in 2019 with Lang et al. [7], who redefined stabilization strategies by exploiting covalent metal–support interactions (CMSIs) independent of defects. By calcining Pt nanoparticles on Fe_2_O_3_ at 800 °C, they achieved atomically dispersed Pt at 1.8 wt% loading. The reducible Fe_2_O_3_ supports trapped mobile PtO_2_ species during calcination, forming thermally stable single atoms. In situ STEM revealed dynamic Pt nanoparticle disintegration into isolated atoms under oxidative conditions, sharply contrasting with irreversible sintering on non-reducible Al_2_O_3_. This work shifted the paradigm from defect-limited anchoring to CMSI-driven stabilization, enabling high metal loadings while maintaining stability. Subsequent studies extended this approach to non-reducible oxides by doping with FeO_x_, broadening the applicability of SACs. Building on these stabilization strategies, researchers turned to microenvironment engineering to enhance catalytic performance. Jiang et al. [8] introduced strain in nanoporous MoS_2_ to amplify synergies between sulfur vacancies (SVs) and single-atom Ru. Tensile strain enriched local reactant density at SVs and optimized Ru’s d-band center, yielding a HER overpotential of 30 mV. Concurrently, Liu et al. [9] demonstrated that curved carbon supports (e.g., onion-like carbon) generated tip-enhanced electric fields, concentrating protons near Pt atoms and achieving a mass activity 43× higher than Pt/C for hydrogen evolution. These studies highlighted the role of geometric and electronic modulation in tailoring active sites, with strain and curvature serving as levers to enhance intrinsic activity.

The latest advancements focus on precise control of surface microenvironments. Zhu et al. [10] engineered tip-like Fe-N_4_ sites on hierarchically porous carbon, combining finite element simulations and in situ spectroscopy to unravel how curvature-induced electric fields densify interfacial water layers and stabilize oxygen intermediates. The catalyst achieved a record ORR half-wave potential of 0.91 V, outperforming Pt/C. This work bridged atomic-scale design with macroscopic performance, illustrating how microenvironment regulation accelerates reaction kinetics. Meanwhile, operando studies revealed dynamic SAC restructuring during methane combustion, where Pt nanoparticles spontaneously dispersed into single atoms under reaction conditions, further validating the stability and adaptability of CMSI-stabilized systems.

Collectively, these milestones reflect a trajectory from foundational stabilization to precision engineering. Early MSI-based strategies addressed aggregation, while CMSIs enabled scalable, defect-independent stabilization. Microenvironment engineering then unlocked enhanced activity through strain, curvature, and field effects.

## 3. Dual-Atom Catalyst

**Figure 2:**
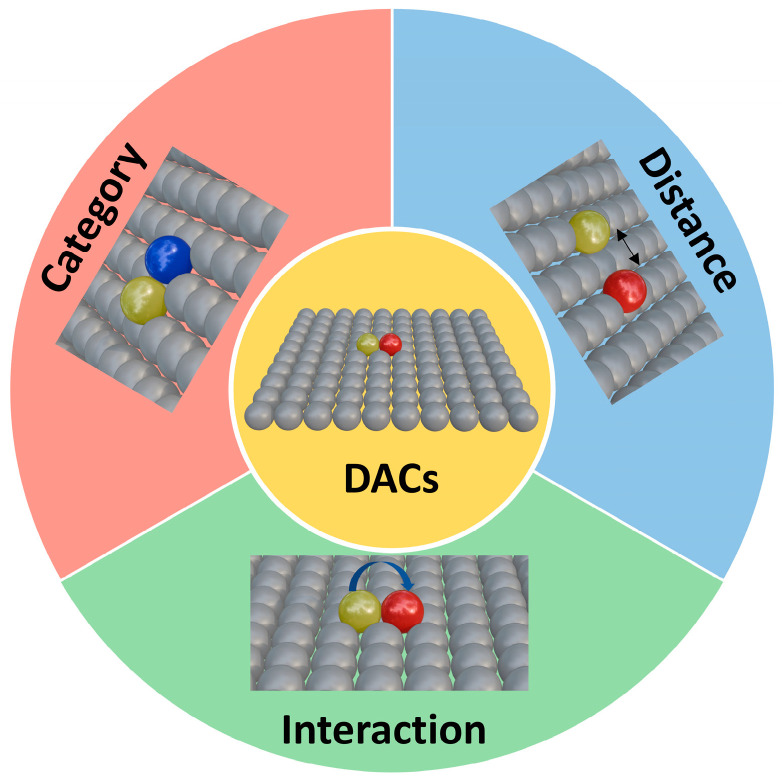
The ‘trinity’ of synergistic effects of atomic categories, distances, and interactions in DACs.

During the development of SACs, researchers observed that SACs face limitations in multi-step electrocatalytic reactions, such as the ORR, HER, and CRR. Since single atomic sites are typically effective only for single-step reactions, SACs have considerable potential for catalytic enhancement. To address these limitations, dual-site catalysts have been developed. Unlike single-atom active sites, dual-site catalysts leverage the synergistic effects of two active sites while retaining atomic-level dispersion, significantly enhancing electrocatalytic activity. The evolution of dual-atom catalysts (DACs) has been a dynamic interplay of atomic composition, spatial configuration, and electronic synergy, with each milestone study refining the understanding of how these factors govern catalytic performance (Figure 2). In 2017, Wang et al. [11] demonstrated the potential of heteronuclear pairs by embedding Fe-Co dual sites in nitrogen-doped carbon derived from Zn/Co bimetallic MOFs. This early work revealed that combining Fe’s high O_2_ affinity with Co’s electron-donating capability reduced the O-O dissociation barrier to 0.25 eV, far lower than single-atom Fe or Co sites. The Fe-Co distance (~2.5 Å) allowed simultaneous O_2_ adsorption across both atoms, splitting the bond via cooperative charge transfer. While the study focused on acidic ORR, it set a precedent for using heteronuclear pairs to break linear scaling relationships, though challenges like thermal stability during synthesis remained unresolved.

By 2019, advances in synthesis precision emerged. Zhang et al. [12] employed atomic layer deposition (ALD) to construct Pt-Ru dimers on nitrogen-doped carbon nanotubes, showcasing how interatomic distance and bonding type could be tailored. The Pt-Ru pairs, spaced at 2.8 Å, exhibited covalent interactions where Ru donated electrons to Pt, weakening Pt’s hydrogen adsorption (ΔG_H* ≈ 0) while retaining Ru’s ability to dissociate H_2_O. This heteronuclear synergy achieved a HER mass activity 54 times higher than Pt/C, with HAADF-STEM and XANES confirming the absence of metallic clusters. The work highlighted the importance of atomic spacing in balancing adsorption and desorption kinetics, as distances below 2.5 Å caused excessive orbital overlap, rigidifying intermediate binding. The subsequent years saw DACs expand into complex reactions like the CO_2_ reduction reaction (CRR). Cheng et al. [13] designed Ni-Cu dual sites within ZIF-8-derived carbon, where Cu’s lower electronegativity enriched Ni’s d-orbital occupancy. At a spacing of ~2.6 Å, the Ni-Cu pair enabled a two-step mechanism: CO_2_ adsorbed on Cu to form *COOH, which then migrated to Ni for C-O bond cleavage. This spatial and electronic division of labor produces 99.2% carbon monoxide selectivity at −0.79 V, which is unattainable with isolated nickel or copper SACs. Concurrently, Leng et al. [14] tackled atomic migration during pyrolysis by encapsulating Fe dimers in polydopamine-coated ZIF-8. The Fe-Fe distance of 2.2 Å, stabilized by N coordination, promoted side-on O_2_ adsorption, accelerating *OOH decomposition and achieving a record ORR half-wave potential of 0.951 V in alkaline media. Operando Raman spectroscopy captured this dynamic, underscoring how precise spacing could stabilize transient intermediates.

The latest breakthroughs focus on unconventional reaction pathways. Liu et al. [15] engineered Ir-Mn dual sites in SrMnO_3_ via ion exchange, shortening the Ir-Mn distance to 2.41 Å (vs. Mn-Mn 2.49 Å). This subtle contraction aligned the spin states of adjacent *O intermediates, enabling direct radical coupling into O_2_ via the oxygen–oxygen radical mechanism (ORCM), bypassing the traditional *OOH pathway. The Ir-Mn interaction shifted Ir’s d-band center downward, reducing overpotentials by 120 mV while maintaining 2000 h stability in PEMWE, a feat attributed to the dual sites’ ability to balance O* adsorption and O_2_ desorption. This study exemplified how atomic type and spacing jointly dictate spin alignment and orbital symmetry, opening avenues for non-precious metal DACs.

Throughout this progression, the interplay of atomic type, distance, and interaction has been pivotal. Early homonuclear pairs (Fe-Fe, Co-Co) established the importance of symmetric adsorption modes, while heteronuclear systems (Pt-Ru, Ni-Cu, Ir-Mn) exploited electronic complementarity to access previously inaccessible pathways. Spatial constraints, whether through MOF templating or ALD precision, ensured optimal interatomic distances that balanced cooperative adsorption with intermediate mobility. Theoretical tools like DFT and machine learning have transitioned from post hoc analysis to predictive design, identifying optimal pairs (e.g., Ir-Mn for ORCM) and guiding synthetic strategies.

## 4. Periodic Single-Metal Site Catalysts

**Figure 3:**
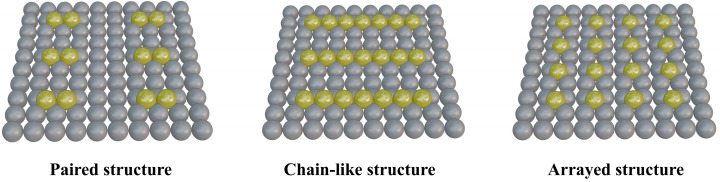
Schematic representation of PSMSCs with different cycle structure types.

Both SACs and DACs contribute to the enhancement of electrocatalytic activity. However, the random distribution of metal atoms creates an inhomogeneous microenvironment at active sites, complicating efforts to fully elucidate catalytic mechanisms and optimize metal atom loading and activity. To address this, researchers have developed PSMSCs, which combine the atomic-level dispersion of SACs with the inter-site synergy of DACs. In PSMSCs, metal atoms are periodically arranged and uniformly aligned, facilitating a clearer understanding of reaction mechanisms. As shown in Figure 3, by arranging metal atoms in ordered configurations—paired, chain-like, or array structures—PSMSCs leverage periodic arrangements to stabilize active sites through geometric and electronic interactions. Paired sites involve adjacent metal atoms enabling cooperative catalysis, chain-type catalysts feature linearly aligned atoms facilitating electron delocalization, and array structures create two-dimensional ordered frameworks for uniform active site distribution. These configurations not only improve catalytic performance but also enhance structural robustness, making them ideal for applications ranging from hydrogen evolution to oxygen reduction reactions.

The development of PSMCs began with the exploration of SACs, with early research focusing on how to stabilize isolated metal atoms and enhance their activity. The low loading density and limited synergistic effects of isolated atoms prompted researchers to turn to more ordered arrangements. The design of periodic structures became possible through innovative synthesis and characterization breakthroughs. For example, Qi et al. [16] engineered Co single-atom arrays on distorted 1T-MoS_2_, where strain-induced phase transitions created metallic channels. The Co atoms, coordinated to sulfur in a chain-like fashion, exhibited near-Pt HER activity due to optimized hydrogen adsorption energies. This study highlighted the critical role of support engineering in stabilizing periodic metal sites. Subsequent landmark studies have extended the design principles: Lunardon et al. [17] systematically reviewed activation strategies for transition metal dichalcogenides (TMDCs), emphasizing how chain-type defects and strain effects in atomic configurations could boost catalytic performance. Concurrently, Sun et al. [18] engineered Co(CN)_3_ microcrystals with well-defined Co-N_3_-C_3_ coordination, creating ordered atomic chains that enhanced oxygen reduction via tailored *OH desorption. These works mark the leap from isolated sites to the ordered arrangement of PSMCs, where spatially periodic electronic structure modulation becomes the key to enhancing the catalytic performance.

With breakthroughs in synthesis techniques, researchers have begun to experiment with the direct construction of atomically ordered metal chains or arrays. Innovations in interfacial engineering have opened up a new path for the scale-up of PSMCs, and the ‘thermal printing’ strategy proposed by Tian et al. [19] enables the temperature-controlled migration of Fe_3_O_4_ nanoparticles to the interface between the SiO_2_ and the carbon layer, which releases Fe atoms and anchors them to the nitrogen–sulfur co-doped carbon skeleton, achieving the precision of the single-atom sites. The precise arrangement of single-atom sites was achieved. This method breaks through the dependence of the traditional loading technique on the distribution of precursors, can flexibly regulate the density of sites, and has been successfully expanded to a variety of metal systems such as Mn, Co, and Pt. The research in this period not only deepened the understanding of the mechanism of periodic structure formation but also verified its practical potential in energy devices such as fuel cells and zinc–air batteries. Moreover, recent advances have focused on precision synthesis and mechanistic elucidation. Zhang et al. [20] modulated electronic metal–support interactions (EMSIs) in Pt/LDH systems using cation vacancies, stabilizing Pt atoms in three oxygen-coordinated configurations that delivered a record turnover frequency (1.3 × 10^5^ h^−1^) for anti-Markovnikov hydrosilylation.

Recent advances in 2024 have further expanded the design principles and mechanistic understanding of PSMSCs. A breakthrough study by Gao et al. [21] demonstrated the confined growth of Co-Ir atomic arrays within graphdiyne (GDY) triangular cavities, achieving ordered dual-metal coordination with an unprecedented mass activity of 2.6 A mg^−1^ for seawater oxidation. The spatial confinement and incomplete charge transfer between GDY and metal atoms stabilized the heteronuclear pairs, enabling synergistic O-O bond activation. Meanwhile, Chang et al. [22] revealed that short-range ordered Ru atom arrays on Co_3_O_4_ (Ruarray-Co_3_O_4_) triggered direct O-O radical coupling through an oxide path mechanism (OPM), bypassing the traditional *OOH intermediate formation. This configuration reduced the OER overpotential to 160 mV in acid while achieving 1500 h stability, addressing the long-standing activity–stability trade-off. Notably, Liu et al. [23] introduced a paradigm-shifting strategy using polarization electric fields (PEFs) on ferroelectric Bi_4_Ti_3_O_12_ nanosheets to align Au atoms into periodic 1D arrays. The Au-O=C=O-Au dual-site adsorption lowered the Gibbs free energy barrier for CO_2_ reduction by 0.48 eV compared to isolated Au sites, while the maintained PEF suppressed charge recombination, achieving a CO production rate 18-fold higher than pristine substrates. These works collectively highlight that ordered atomic arrangements not only optimize intermediate adsorption through cooperative dual-site effects but also stabilize catalytic matrices by suppressing depolarization.

In summary, the progression from isolated SACs to ordered PSMSCs marks a paradigm shift in catalysis. By leveraging periodic arrangements and advanced synthesis techniques, researchers have unlocked unprecedented control over active site geometry, stability, and electronic properties. Key milestones include the stabilization of single atoms through EMSI, the exploitation of defects for site activation, and innovative methods like thermal printing for interfacial atomic placement. Recent breakthroughs, including graphdiyne-confined heteronuclear arrays, acid-stable Ru OPM catalysts, and polarization-engineered 1D Au ensembles, have demonstrated that periodic atomic arrangements unlock unconventional reaction pathways (e.g., radical coupling mechanisms) while resisting metal dissolution. Key advances involve precise spatial control through confinement effects or external fields, which balance orbital hybridization for optimal intermediate binding. The emergence of operando techniques has further illuminated dynamic restructuring under operational conditions, enabling rational design rules for next-generation PSMSCs. Future directions may focus on expanding these strategies to diverse metal–support systems, elucidating dynamic structural changes under operando conditions, and integrating computational models to predict optimal configurations. As the field advances, PSMSCs hold immense potential to bridge the gap between homogeneous and heterogeneous catalysis, offering sustainable solutions for energy conversion and chemical synthesis.

The evolution from SACs to DACs and ultimately PSMSCs reflects a strategic progression toward enhanced activity, stability, and mechanistic clarity. To systematically compare their performance, we summarize key metrics across representative reactions (ORR, HER, and CRR) in Table 1.

## 5. Conclusions

This paper provides a comprehensive exploration of the development pathway of ADCs, emphasizing their critical role in advancing electrocatalytic processes essential for sustainable energy conversion. Through the examination of SACs and DACs, we demonstrate the significant advantages these systems offer, including high atom utilization, enhanced catalytic activity, and improved thermal stability. The innovative methods discussed, such as co-deposition and atomic layer deposition, enable precise control over atomic configuration, ultimately optimizing catalytic performance. Furthermore, the introduction of PSMSCs represents a substantial advancement in catalyst design, addressing challenges posed by the random distribution of metal atoms. This new class of catalysts enhances our understanding of reaction mechanisms while achieving uniform metal site loading, thereby improving overall electrocatalytic activity.

The remarkable evolution of ADCs, from SACs and DACs to PSMSCs, has redefined electrocatalysis by merging atomic precision with the robustness of heterogeneous systems. SACs maximize metal utilization and mechanistic transparency, while DACs overcome multi-step reaction barriers through synergistic dual-site interactions, and PSMSCs introduce ordered atomic ensembles to unify activity, stability, and uniformity. These advancements, driven by innovations in covalent metal–support interactions (CMSIs), microenvironment engineering, and operando characterization, have illuminated dynamic atomic restructuring under reaction conditions, enabling rational design principles for next-generation catalysts.

Beyond the current ADC architectures, three emerging nanomaterials present transformative opportunities for catalyzing industrially relevant reactions. Thiolated gold clusters (Au_n_(SR)_m_) exhibit atomic precision in ligand engineering, enabling tailored electronic structures for catalytic applications. For instance, Au_25_(SR)_18_ demonstrates dynamic control over O_2_ activation through ligand-mediated charge transfer, offering a model system for probing metal–ligand cooperativity in redox reactions [27]. Covalent Organic Frameworks (COFs) further bridge the gap between molecular and heterogeneous catalysis by embedding single-atom sites (e.g., Cu_SA_/COFs) within periodic porous networks. The crystalline architecture of COFs enhances exciton dissociation efficiency, as shown in their ability to degrade emerging organic contaminants via photo-Fenton-like mechanisms with a 39.5-fold increase in reaction kinetics compared to conventional systems [28]. Most intriguingly, volleyballene-type nanostructures (e.g., Sc_8_C_48_N_12_) introduce a novel paradigm by integrating transition metals into carbide frameworks. These metalloid cages selectively dissociate NO_x_ pollutants through synergistic Sc 3d orbital polarization and carbide-mediated intermediate stabilization, achieving bond cleavage without energy barriers, a critical advantage for industrial-scale pollution mitigation [29]. Collectively, these systems redefine catalytic design through atomic-level control over active sites, electronic modulation, and self-supporting architectures.

However, the complexity of atomic-level interactions and the need for scalable synthesis demand a paradigm shift toward data-driven approaches, where artificial intelligence (AI) and machine learning (ML) emerge as transformative tools. The integration of AI/ML with catalysis research holds immense potential to accelerate discovery and optimization. Predictive models trained on high-throughput density functional theory (DFT) datasets can identify optimal atomic configurations, such as heteronuclear DAC pairs or PSMSC arrays, by forecasting adsorption energies, d-band centers, and stability metrics. For instance, generative adversarial networks (GANs) could propose novel Ir-Mn DAC configurations that bypass traditional oxygen evolution pathways. Beyond static design, AI-enhanced operando characterization techniques, such as real-time scanning transmission electron microscopy (STEM) or X-ray absorption spectroscopy (XAS), can decode transient intermediates and atomic migration dynamics. Neural networks analyzing time-resolved spectral data may uncover hidden correlations between microenvironment shifts (e.g., curvature-induced electric fields) and catalytic performance, enabling adaptive control of reaction conditions to maintain peak efficiency.

Synthesis challenges, particularly in scaling PSMSCs, could be mitigated through reinforcement learning (RL) algorithms that optimize parameters like pyrolysis temperatures or precursor ratios. Reinforcement learning (RL) algorithms can guide synthetic protocols (e.g., ALD or thermal printing) by optimizing parameters such as temperature, precursor ratios, and support defects. Additionally, natural language processing (NLP) tools mining vast catalysis literature could unearth overlooked design principles, while graph neural networks (GNNs) modeling atomic interactions in SACs/DACs might reveal non-intuitive structure–activity relationships. For example, ML-assisted analysis of electronic metal–support interactions (EMSIs) in Pt/LDH systems could generalize stabilization strategies across diverse metal–support combinations, reducing reliance on trial-and-error experimentation.

Despite these prospects, critical challenges persist. Standardized datasets for ADC properties (e.g., coordination environments, stability thresholds) are urgently needed to train robust ML models. Additionally, the integration of AI with automated synthesis platforms (e.g., robotic ALD or inkjet printing) remains in its infancy but holds transformative potential for high-throughput ADC fabrication. Ethical considerations, including energy costs of ML training and equitable access to computational resources, must also be addressed to ensure sustainable innovation.

The ADC field stands at a crossroads, where atomic-level precision meets data-driven discovery. By synergizing AI/ML with advanced synthesis, characterization, and theory, researchers can unlock catalysts with programmable microenvironments, self-optimizing active sites, and industrial-scale durability. These efforts will not only advance fundamental catalysis science but also accelerate the global transition to renewable energy systems, positioning ADCs as cornerstones of a carbon-neutral future.

## Figures and Tables

**Table 1:** Performance comparison of SACs, DACs, and PSMSCs in selected electrocatalytic reactions.

Property	SACs	DACs	PSMCs
Active Site Density	High	Moderate	High
HER (Overpotential)	Ru/np-MoS_2_ [8] (30 mV @ 10 mA cm^−2^)	CoNi-Ti_3_C_2_T_x_ MXene [24] (31 mV @ 10 mA cm^−2^)	SA Co-D 1T MoS_2_ [16] (42 mV @ 10 mA cm^−2^)
ORR (Half-wave potential)	T-FeSAC [10] (0.91 V vs. RHE)	Fe_2_@PDA-ZIF-900 [14] (0.951 V vs. RHE)	Fe SAs/S-NC [19] (0.91 V vs. RHE)
CRR (Faradaic Efficiency CO_2_ to CO)	C–NiN_4_-1000 [25] (96.11%)	Ni/Cu-N-C [13] (99.2%)	Ni-SAC-NA [26] (96.7%)
Scalability	Challenging	Moderate	High
